# Lymph node density as a prognostic variable in node-positive bladder cancer: a meta-analysis

**DOI:** 10.1186/s12885-015-1448-x

**Published:** 2015-06-02

**Authors:** Ja Hyeon Ku, Minyong Kang, Hyung Suk Kim, Chang Wook Jeong, Cheol Kwak, Hyeon Hoe Kim

**Affiliations:** 1 Department of Urology, Seoul National University Hospital, Seoul, Republic of Korea; 2 Department of Urology, Seoul National University Bundang Hospital, Seongnam City, Kyeonggi-do Republic of Korea

**Keywords:** Bladder cancer, Meta-analysis, Lymph node density, Prognosis, Radical cystectomy

## Abstract

**Background:**

Although lymph node (LN) status and the LN burden determine the outcome of bladder cancer patients treated with cystectomy, compelling arguments have been made for the incorporation of LN density into the current staging system. Here, we investigate the relationship between LN density and clinical outcome in patients with LN-positive disease, following radical cystectomy for bladder cancer.

**Methods:**

PubMed, SCOPUS, the Institute for Scientific Information Web of Science, and the Cochrane Library were searched to identify relevant published literature.

**Results:**

Fourteen studies were included in the meta-analysis, with a total number of 3311 patients. Of these 14 publications, 6 studies, (533 patients), 10 studies (2966 patients), and 5 studies (1108 patients) investigated the prognostic association of LN density with disease-free survival (DFS), disease-specific survival (DSS), and overall survival (OS), respectively. The pooled hazard ratio (HR) for DFS was 1.45 (95 % confidence interval [CI], 1.10–1.91) without heterogeneity (I^2^ = 0 %, p = 0.52). Higher LN density was significantly associated with poor DSS (pooled HR, 1.53; 95 % CI, 1.23–1.89). However, significant heterogeneity was found between studies (I^2^ = 66 %, p = 0.002). The pooled HR for OS was statistically significant (pooled HR, 1.45; 95 % CI, 1.11–1.90) without heterogeneity (I^2^ = 42 %, p = 0.14). The results of the Begg and Egger tests suggested that publication bias was not evident in this meta-analysis.

**Conclusions:**

The data from this meta-analysis indicate that LN density is an independent predictor of clinical outcome in LN-positive patients. LN density may be useful in future staging systems, thus allowing better prognostic classification of LN-positive bladder cancer.

**Electronic supplementary material:**

The online version of this article (doi:10.1186/s12885-015-1448-x) contains supplementary material, which is available to authorized users.

## Background

Radical cystectomy with lymph node (LN) dissection remains the standard treatment for patients with muscle-invasive urothelial carcinoma of the bladder, and also for non-muscle-invasive disease, refractory to intravesical therapy. Pelvic LN involvement occurs in approximately 25 % of patients undergoing radical cystectomy for urothelial cancer [1]; when LN positivity is observed, the 10-year mortality rate can reach 80 %, despite adjuvant chemotherapy [2, 3]. Although LN involvement portends a relatively poor prognosis, some patients exhibit long-term survival following surgery, with, or without systemic chemotherapy [4]. Efforts have been made to stratify LN-positive patients according to different prognostic factors to obtain more individualized risk estimations. Although several prognostic factors have previously been reported for LN-positive patients, predictive factors for survival in LN-positive patients have not been clearly defined.

The concept of LN density, i.e. the number of LNs containing metastatic deposits divided by the total number of LNs removed, was first described for bladder cancer in 2003 [5, 6]. Recent studies have suggested that LN density is superior to the tumor-node-metastasis (TNM) classification system [5], and to the absolute number of positive LNs [5, 7] in predicting disease-free survival (DFS) and disease-specific survival (DSS). Although radical surgery alone cures 5–34 % of patients with LN-positive disease, most survivors have only 1–2 microscopically involved LNs, rather than grossly positive, or multiple LN involvement [8]. Therefore, LN metastasis (LN status), and the number of involved LNs (LN burden) determine the outcome of patients with bladder cancer treated with cystectomy [8]. Compelling arguments have been made for the incorporation of LN density into the current American Joint Committee on Cancer (AJCC) staging system [9]. The present study aimed to elucidate the relationship between LN density and clinical outcome in LN-positive patients with bladder cancer following radical cystectomy.

## Methods

This analysis was conducted in accordance with the Preferred Reporting Items for Systematic Reviews and Meta-Analyses (PRISMA) guidelines (Additional file 1) [10].

### Data sources and search strategy

PubMed, SCOPUS, the Institute for Scientific Information Web of Science, and the Cochrane Library were searched to identify potentially relevant published literature. The search was performed in August 2014. The search terms used included “bladder cancer,” “radical cystectomy,” and “lymph node density.” We also carefully examined the references of articles and reviews to identify potential additional studies.

### Study eligibility

Studies were eligible for inclusion in the meta-analysis if they met the following criteria: (1) patients studied had LN-positive bladder cancer; (2) LN density was measured; (3) the association between LN density and clinical outcome was investigated; and (4) the full text articles were published in English. Studies were excluded based on the following criteria: (1) if they were abstracts, review articles, case reports, letters, or laboratory studies; (2) if key information for further analysis was absent; (3) when part, or all, of the same patient series was included in more than one publication, the largest sample size, or the most recent publication was included to avoid duplication of the same survival data; and (4) when studies did not report an adjusted hazard ratio (HR) in multivariate analysis, as the accuracy of HRs without using multivariate analysis is uncertain. However, if the result was negative in univariate analysis and as a result, LN density could not be included in multivariate analysis, the result of the univariate analysis was included. Two reviewers (MK and HSK) independently determined study eligibility. Disagreements were resolved by consensus.

### Data extraction and quality assessments

Using a standardized form, data extraction from each of the included studies was performed independently by two reviewers (CK and CWJ). When discrepancies arose between two reviewers, discussion with another reviewer (HHK) was undertaken until a consensus was reached. Quality assessment in this meta-analysis was carried out using the REporting recommendations for tumor MARKer prognostic studies (REMARK) guidelines and quality scale [11, 12], and included the following study parameters: (1) inclusion and exclusion criteria; (2) prospective or retrospective data; (3) sufficient description of patient and tumor characteristics; (4) sufficient description of LN density measurement; (5) well-defined study endpoint; (6) description of patient follow-up period; and (7) identification of patients lost to follow-up or not available for statistical analysis. Scores ranged from 0 to 8; studies with a total score of 8 were considered to show the highest study quality, whereas a score of 0 indicated studies with the lowest quality.

### Statistical analysis

We calculated the pooled HR with its corresponding 95 % confidence interval (CI) to assess the association of LN density with survival in LN-positive patients. A HR of >1 indicated a worse prognosis in patients with higher LN density, if the 95 % CI did not overlap. If explicit survival data were not provided, they were calculated from the available numerical data using methods reported by Parmer et al. [13]. A meta-analysis was performed using the DerSimonian and Laird random effects model, applying the inverse of variance as a weighing factor [14]. Heterogeneity between studies was estimated by using the Cochran Q-static and I^2^ tests [15]. A Q-test with a p-value of <0.05 or an I^2^ value of >50 % was considered to represent substantial heterogeneity between studies. We also used subgroup analysis with meta-regression analysis to explore the sources of heterogeneity. Funnel plots, the Begg rank correlation test, and the Egger linear regression test were applied to explore potential publication bias, and a p-value of <0.05 was considered significant [16, 17]. All statistical tests were two-sided, and statistical significance was defined as p < 0.05. RevMan statistical software version 5.0 (the Cochrane Collaboration, Copenhagen, Denmark) was used in this study. Meta-regression and publication bias were analyzed using R statistical software version 2.13.0 (R development Core Team, Vienna, Austria; http://www.r-project.org).

## Results

**Fig. 1:**
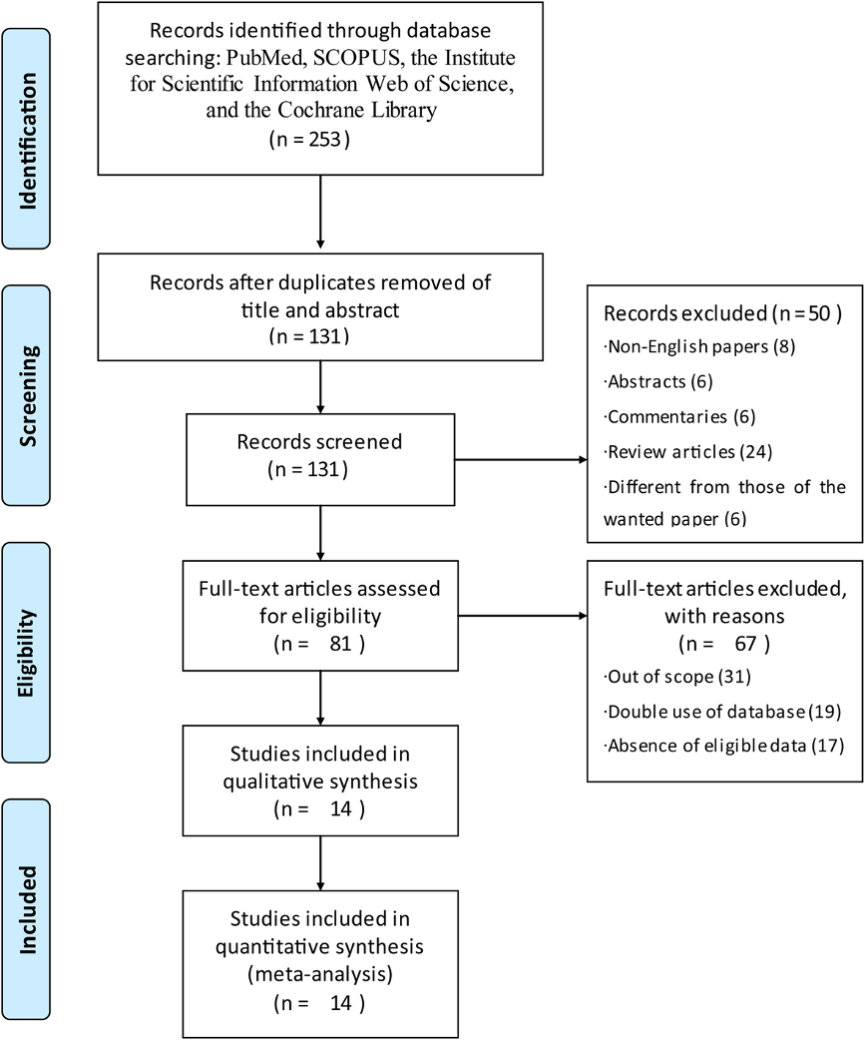
Flow chart of the literature search used in this meta-analysis

The search strategy retrieved 253 publications, of which 81 were reviewed for eligibility, with 14 studies finally included [5, 18–30]. The detailed screening process used is shown in Fig. 1.

### Study characteristics

The characteristics of the selected studies are described in Table 1. The total number of patients from all of the studies was 3311 (range, 43–1038; median, 93). The included studies were published between 2003 and 2014. Three studies were conducted in Asian countries, and 11 studies were carried out in non-Asian countries. Among these 14 studies, although data were collected prospectively in 4 studies, none of selected studies was prospective study. Different cut-off values were used for LN density. The quality scores ranged from 3 to 6. As shown in Table 1, 10 of the 14 studies had quality scores of <5, suggesting that most of the studies were not well designed. Other characteristics of the eligible studies are reported in Tables 2 and 3.

**Table 1:** Main characteristics of the eligible studies

Study	Year	Country	Recruitment period	Prospective data collection	Inclusion and exclusion criteria	Definition of survival	Definition of LN density	Cut-off of LN density	Interpretation of LN density	Quality scale
Herr [5]	2003	USA	1979–1999	No	No	No	Yes	20	NA	3
Fleischmann [18]	2005	Switzerland	1985–2000	Yes	Yes	Yes	No	20	NA	5
Osawa [19]	2009	Japan	1990–2005	No	Yes	No	No	25	NA	4
Wiesner [20]	2009	Germany	2001–2006	Yes	No	No	No	11	NA	3
Furukawa [21]	2010	Japan	1995–2003	No	Yes	No	No	25	NA	4
Guzzo [22]	2010	USA	1988–2003	Yes	Yes	No	Yes	25	NA	5
Stephenson [23]	2010	USA	1999–2007	No	No	No	No	20	Blind	3
May [24]	2011	Germany	1989–2008	No	Yes	No	Yes	20	NA	5
Jensen [25]	2012	Denmark	2004–2009	Yes	Yes	Yes	Yes	10	NA	6
Morgan [26]	2012	USA	1992–2006	No	Yes	Yes	Yes	20	NA	4
Kassouf [27]	2013	Multination	1993–2005	No	Yes	Yes	No	None*	NA	4
Masson-Lecomte [28]	2013	France	2002–2011	No	No	No	Yes	20	NA	4
Mmeje [29]	2013	USA	2005–2009	No	No	Yes	Yes	20	NA	4
Kwon [30]	2014	Korea	1990–2011	No	Yes	Yes	No	18	NA	4

LND: lymph node density, NA: not available

**Table 2:** Patient characteristics from the eligible studies

Study	No. of patients	Median age, range (years)	Gender (male/female)	Upper limit of PLND	Neoadjuvant chemotherapy	Adjuvant chemotherapy	Median Follow-up, range (months)
Herr [5]	162	67 (36–87)	NA	Distal common iliac artery	0	NA	90 (24–180)
Fleischmann [18]	101	67 (35–89)	87/14	Crossing of the ureter with common iliac artery	0	41	21 (1–191)
Osawa [19]	60	68 (34–84)	48/12	Below the bifurcation of common iliac artery (almost) Above iliac bifurcation (a few)	0	25	41 (4–138)
Wiesner [20]	46	NA	NA	Inferior mesenteric artery	0	27	22 (1–76)
Furukawa [21]	82	70.3 (42–86)	62/20	Distal common iliac artery	0	17	33.6 (mean) (2–142)
Guzzo [22]	85	NA	67/18	Bifurcation of common iliac artery	0	55	46 (3–223)
Stephenson [23]	134	68 (IQR: 59–75)	NA	Distal common iliac artery	0	90	23 (IQR: 10–36)
May [24]	477	66.3 (33–86)	376/101	NA	0	159	16
Jensen [25]	43	NA	NA	Inferior mesenteric artery	0	0	53 (24–83)
Morgan [26]	779	NA	530/249	NA	28	296	NA
Kassouf [27]	1,038	67 (IQR: 60–73)	821/217	Not standardized	0	NA	33 (IQR: 14–69)
Masson-Lecomte [28]	75	65 (31–85)	64/11	Common iliac bifurcation	NA	46	40.6 (3–127)
Mmeje [29]	50	69 (mean) (50–83)	38/12	Aortic bifurcation	29	19	39.6 (16–75)
Kwon [30]	179	NA	NA	Not standardized	0	NA	64.3 (1–231.4)

PLND: pelvic lymph node dissection, NA: not available, IQR: interquartile range

**Table 3:** Pathologic characteristics from the eligible studies

Study	Tumor grade (G0/G1/G2/G3)	Pathologic T stage (pT0/is/a/1/2/3/4)	Pathologic N stage (pN1/2/3)	Median no. of LNs removed, range	Median no. of positive LNs, range	Median LN density, range (%)
Herr [5]	NA	79 (≤T2)/123/0	54/87/21	13 (2–32)	3.3	NA
Fleischmann [18]	NA	0/0/0/19 (T1/2)/53/30	32/69/0	22 (10–43)	NA	NA
Osawa [19]	0/0/9/51	0/0/0/1/6/38/15	21/39	12 (1–80)	2 (1–12)	23.1 (1.3-100)
Wiesner [20]	0/0/8/38	0/0/0/3/11/24/8	NA	33 (15–77)	3 (1–28)	11 (1–73)
Furukawa [21]	0/0/12/70	0/0/0/0/19/37/26	32/50/0	14.4 (mean) (6–37)	3.1 (mean) (1–12)	25.3 (2.8–100)
Guzzo [22]	NA	9 (≤T1)/13/63 (T3/4)	NA	16.7 (mean) (5–56)	NA	NA
Stephenson [23]	NA	107 (≤T2)/27 (T3/4)	62/72 (N2/3)	14 (IQR: 9–20)	2 (IQR: 1–3)	17 (IQR: 9–38)
May [24]	79 (≤G2)/398	24 (≤T1)/103/350 (T3/4)	187/290/0	12 (1–66)	2 (1–25)	17.6 (2.3–100)
Jensen [25]	NA	NA	16/9/18	NA	NA	NA
Morgan [26]	27 (LG)/741 (HG)	14 (≤T1)/48/131/585	NA	9 (IQR: 4–16)	2 (IQR: 1–3)	25 (IQR: 13–50)
Kassouf [27]	NA	65 (≤T1)/176/505/292	NA	18 (IQR: 11–32)	2 (IQR: 1–5)	14.3 (IQR: 6.7–33.3)
Masson-Lecomte [28]	NA	0/0/0/2/15/39/19	10/10/6	18 (3–49)	3 (1–35)	19 (2–100)
Mmeje [29]	NA	0/1/0/2/13/26/7	NA	19 (mean) (5–35)	3 (mean) (1–12)	NA
Kwon [30]	NA	NA	62/116/1	16 (1–118)	3 (1–37)	17.6 (2.6–100)

LV: lymph node, NA: not available, IQR: interquartile range, LG: low grade, HG: high grade

### Outcomes from eligible studies

Of the 14 publications included in the meta-analysis, 6 studies (533 patients), 10 studies (2966 patients), and 5 studies (1108 patients) investigated the prognostic association of LN density with DFS, DSS, and overall survival (OS), respectively (Tables 3, 4, 5, and 6).

**Table 4:** Estimation of the hazard ratio for disease-free survival

Study	HR estimation	Co-factors	Analysis results
Fleischmann [18]	HR, 95 % CI	Extracapsular extension, no. of positive LNs	Not significant
Guzzo [22]	HR, 95 % CI	Age, sex, diversion type, pT stage, adjuvant chemotherapy	Not significant
Jensen [25]	HR, 95 % CI	Age, sex, pT stage, pN stage, metasis above the aortic bifurcation, extracapsular extension, volume dependent LN density, diameter of largest LN, volume of metastatic LNs	Not significant
Masson-Lecomte [28]	HR, 95 % CI	pT stage, lymphovascular invasion, extracapsular extension, adjuvant chemoethrapy	Significant
Mmeje [29]	P value, event no. (univariate)	-	Not significant
Kwon [30]	HR, 95 % CI	pT stage, pN stage, no. of positive LNs, adjuvant chemotherapy	Not significant

HR: hazard ratio, CI: confidence interval, LN: lymph node

**Table 5:** Estimation of the hazard ratio for disease-specific survival

Study	HR estimation	Co-factors	Analysis results
Herr [5]	P value, event no.	pT stage, pN stage, no. of LNs removed, no. of positive LNs	Significant
Wiesner [20]	HR, 95 % CI	No. of LNs removed, no. of positive LNs	Significant
Furukawa [21]	HR, 95 % CI	No. of positive LNs, laterality of positive LNs, adjuvant chemotherapy	Significant
Guzzo [22]	HR, 95 % CI	Age, sex, diversion type, pT stage, adjuvant chemotherapy	Not significant
May [24]	HR, 95 % CI	Age, sex, radical cystectomy time frame, pT stage, pN stage, tumor grade, concomitant carcinoma in situ, adjuvant chemotherapy, no. of LNs removed	Significant
Jensen [25]	HR, 95 % CI	Age, sex, pT stage, pN stage, metastasis above the aortic bifurcation, extracapsular extension, volume dependent LN density, diameter of largest LN, volume of metastatic LNs	Not significant
Morgan [26]	HR, 95 % CI	Age, Charlson comorbidity index, pT stage, tumor grade, no. of LNs removed, adjuvant chemotherapy, diversion type, year of surgery, surgeon volume, transfusion	Significant
Kassouf [27]	HR, 95 % CI	Age, sex, tumor grade, pT stage, margin status, lymphovascular invasion, adjuvant chemotherapy, concomitant carcinoma in situ	Significant
Masson-Lecomte [28]	P value, event no.	pT stage, extracapsular extension, adjuvant chemoethrapy	Not significant
Kwon [30]	HR, 95 % CI	pT stage, pN stage, no. of positive LNs, adjuvant chemotherapy	Not significant

HR: hazard ratio, LN: lymph node, CI: confidence interval

**Table 6:** Estimation of the hazard ratio for overall survival

Study	HR estimation	Co-factors	Analysis results
Osawa [19]	HR, 95 % CI	Histology, no. of positive LNs, adjuvant chemotherapy	Significant
Guzzo [22]	HR, 95 % CI	Age, sex, diversion type, pT stage, adjuvant chemotherapy	Not significant
Stephenson [23]	HR, 95 % CI	pT stage, aggregate LN metastasis diameter, lymphovascular invasion, margin status, extracapsular extension	Not significant
Morgan [26]	HR, 95 % CI	Age, Charlson comorbidity index, pT stage, tumor grade, no. of LNs removed, adjuvant chemotherapy, diversion type, year of surgery, surgeon volume, transfusion	Significant
Mmeje [29]	P value, event no. (univariate)	-	Not significant

HR: hazard ratio, CI: confidence interval, LN: lymph node

**Fig. 2:**
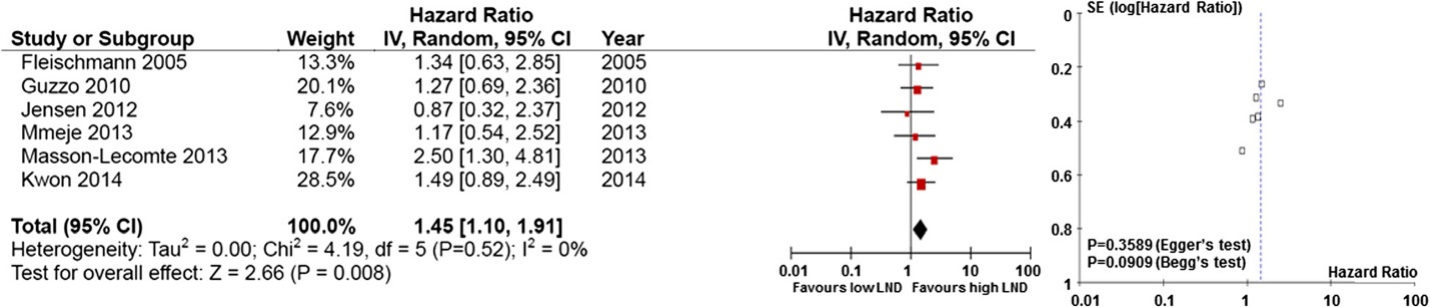
Forest plots of disease-free survival by lymph node density. (Left) The horizontal lines correspond to the study-specific hazard ratio (HR) and 95 % confidence interval (CI), respectively. The area of the squares reflects the study-specific weight. The diamond represents the pooled results of HR and 95 % CI. (Right) The Begg test funnel plots for publication bias. Each point represents a separate study of the indicated association. The vertical line represents the mean effects size

**Fig. 3:**
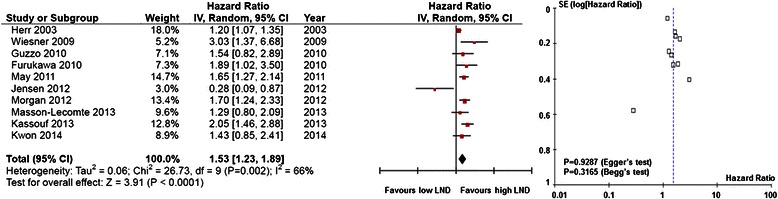
Forest plots of disease-specific survival by lymph node density. (Left) The horizontal lines correspond to the study-specific hazard ratio (HR) and 95 % confidence interval (CI), respectively. The area of the squares reflects the study-specific weight. The diamond represents the pooled results of HR and 95 % CI. (Right) The Begg test funnel plots for publication bias. Each point represents a separate study of the indicated association. The vertical line represents the mean effects size.

**Fig. 4:**
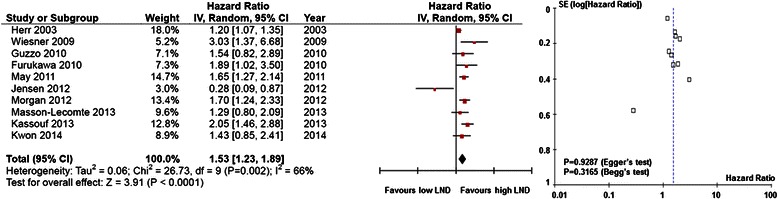
Forest plots of overall survival by lymph node density. (Left) The horizontal lines correspond to the study-specific hazard ratio (HR) and 95 % confidence interval (CI), respectively. The area of the squares reflects the study-specific weight. The diamond represents the pooled results of HR and 95 % CI. (Right) The Begg test funnel plots for publication bias. Each point represents a separate study of the indicated association. The vertical line represents the mean effects size

The results of the meta-analysis are shown in Figs. 2, 3, and 4. Overall, the pooled HR for DFS was 1.45 (95 % CI, 1.10–1.91), suggesting that a higher LN density was an indicator of poor prognosis for bladder cancer. No significant heterogeneity was observed among the studies (I^2^ = 0 %, p = 0.52) (Fig. 2). A meta-analysis of 10 studies found that higher LN density was significantly associated with poor DSS (pooled HR, 1.53; 95 % CI, 1.23–1.89). However, significant heterogeneity was found between studies (I^2^ = 66 %, p = 0.002) (Fig. 3). Subgroup analysis with meta-regression analysis showed that the number of patients (p_heterogeneity_ = 0.0015), median follow-up (p_heterogeneity_ = 0.0017), and quality scale (p_heterogeneity_ = 0.0233) were possible explanations for heterogeneity (Table 7). Meta-analysis of the 5 studies evaluating the association of LN density with OS found that a higher LN density predicted a worse outcome, with a pooled HR of 1.45 (95 % CI, 1.11–1.90). Inter-study heterogeneity was not significant (I^2^ = 42 %, p = 0.14) (Fig. 4).

**Table 7:** Subgroup analysis for disease-specific survival

	No. of included articles	No. of cases	Pooled HR (95 % CI)	Chi^2^ (p value)	I^2^	P_h_^*^
Publication year						0.0517
2003–2010	4	375	1.61 (1.09–2.38)	7.42 (0.06)	60 %	
2011–2014	6	2591	1.51 (1.16–1.97)	12.22 (0.03)	59 %	
Region						0.3206
USA	3	1979	1.57 (1.10–2.22)	11.45 (0.003)	83 %	
Europe	5	1679	1.54 (1.02–2.31)	14.19 (0.007)	72 %	
Asia	2	261	1.61 (1.08–2.39)	0.46 (0.5)	0 %	
No. of patients						0.0015
<100	5	331	1.40 (0.82–2.40)	12.35 (0.01)	68 %	
≥100	5	2635	1.55 (1.23–1.95)	14.06 (0.007)	72 %	
Median follow-up*						0.0017
<36 months	4	1643	1.85 (1.53–2.24)	2.58 (0.46)	0 %	
≥36 months	5	544	1.20 (0.91–1.59)	7.43 (0.11)	46 %	
Analysis results						0.1626
Not significant	4	382	1.14 (0.69–1.87)	7.37 (0.06)	59 %	
Significant	6	2584	1.69 (1.31–2.17)	18.98 (0.002)	74 %	
Quality scale						0.0233
≤4	7	2361	1.60 (1.26–2.03)	16.99 (0.009)	65 %	
>4	3	605	1.08 (0.51–2.30)	8.91 (0.01)	78 %	

HR: hazard ratio, CI: confidence interval

P_h_^*^ for heterogeneity between subgroups with meta-regression analysis

*One study was excluded because the duration of follow-up was not available (Morgan [26])

### Publication bias

No obvious asymmetry was evident in the Funnel plots of any contrast (Figs. 2, 3, and 4). All the p-values for the Begg and Egger tests for DFS, DSS, and OS were >0.05, providing statistical evidence of funnel plots’ symmetry. These results suggest that publication bias was not evident in this meta-analysis.

## Discussion

Up to 25 % of clinically organ-confined tumors show evidence of LN metastasis at the time of surgery. Pathologic specimens from contemporary radical cystectomy series reveal that the rate of LN metastasis increases from 5 % in non-muscle-invasive bladder tumors (≤pT1), to 18 % in pT2a, 27 % in pT2b, and 45 % in pT3–4 [2]. Although LN-positivity is an adverse prognostic factor per se, some LN-positive patients experience long-term survival following radical cystectomy. Therefore, LN dissection may be curative in a selected subset of LN-positive patients [18]. However, prognostic criteria to identify this population have not been defined.

Several prognostic factors have previously been reported for LN-positive patients: (1) pathologic stage of the primary tumor [6, 31]; (2) presence of lymphovascular invasion of the primary tumor [18]; (3) pN stage using the TNM classification; (4) number of LNs involved [2,618,20,32]; (5) number of LNs removed at cystectomy [33–35]; (6) LN density [5, 6]; and (7) the presence of extracapsular extension [18, 28, 36]. However, factors predictive of survival in LN-positive patients are debated.

The pT stage of the TNM classification remains significant in LN-positive bladder cancer [6, 31]. Although differentiation between pT2 and pT3 disease seems unnecessary when LN invasion is present, Stein et al. [6] have previously shown the prognostic significance of extravesical tumor extension compared to organ-confined tumor in LN-positive patients. However, the prognostic significance of the pN stage is unclear [5, 28], although risk stratification of recurrence and survival following radical cystectomy has traditionally been based on TNM staging. The accuracy of the most recent TNM staging system has also been questioned [37, 38], as the location of positive LNs does not seem to have prognostic significance. The number of positive LNs appears to be a significant adverse prognostic factor. Some studies have demonstrated decreased DFS and OS associated with an increasing absolute number of positive LNs [2, 6, 18, 20, 32], but not all studies have confirmed these findings. In addition, the cut-off number for positive LNs that influence outcome is controversial. Furthermore, the total number of positive LNs does not reflect the tumor burden, and its significance is influenced by the extent of the LN dissection. Other studies have demonstrated that the total number of LNs removed, irrespective of LN positivity, is a significant prognostic factor [6, 33–35]. Extracapsular extension may be an independent prognostic factor for DFS and DSS in LN-positive bladder cancer and upper urothelial carcinoma [18, 28, 36].

It has been suggested that LN density is more useful in stratifying patients with LN-positive bladder cancer. Herr [5] found that a LN density cut-off of 20 % was superior to the most recent TNM staging system in predicting DSS and local recurrence, on multivariate analysis. Stephenson et al. [23] also suggested that the aggregate LN metastasis diameter, LN density, and extranodal extension should be considered as the novel predictors in a revised TNM-staging system. However, despite the attempts of multiple studies to explore the association between LN density and its potential association with disease recurrence or death, the results have been inconsistent. For example, none of the new LN-dependent markers, such as localization within the pelvic cavity, extracapsular extension, and LN density were independently significant in the prospective study by Jenson et al. [25]. To our knowledge, the present meta-analysis is the first to clarify the association between LN density and survival in LN-positive bladder cancer using meta-analysis and systematic review. In this meta-analysis, studies reporting HRs of cumulative survival rates were qualitatively summarized using standard meta-analysis techniques. Fourteen studies, with a total of 3311 LN-positive patients, stratifying DFS, DSS, and/or OS by LN density were eligible for inclusion in the meta-analysis. Higher LN density was independently associated with poorer DFS, DSS, and OS. As our meta-analysis includes 14 eligible studies, with a total of 3311 patients, it provides stronger statistical power and a more precise estimation of results than previously published reports. Moreover, our meta-analysis was mainly based on adjusted estimates, and statistical significance was observed for all three end-points, DFS, DSS, and OS.

However, to reach a convincing conclusion regarding the value of LN density for the prognosis of LN-positive bladder cancer, some issues should also be addressed. First, we considered that the definition of what constitutes a “lymph node” varies among urological pathologists in different series. This can impact the nodal yields, and therefore, the burden of lymph node density. Second, the cut-off points for LN density were arbitrarily determined retrospectively, and they have not been validated sufficiently in alternative data sets [8]. Therefore, the threshold for clinically relevant LN density varies between multiple studies and has yet to be established. Third, there is no prospectively evaluated standardized template for pelvic LN dissection. Some data support the use of LN density rather than the absolute number of positive LNs when extended pelvic LN dissection is performed [27]. On the contrary, LN density may be a less sensitive determinant of outcome following limited dissection [8]. Additionally, there were no surgical consistency and uniformity of techniques between previous studies. Therefore, different LN dissection templates, and different surgical procedures may contribute significant bias to a meaningful analysis. Fourth, the number of LNs removed may affect the value of LN density. Jeong et al. [39] demonstrated that when more than 15 LNs were removed, LN density was a predictive factor for survival. In a report by Kassouf et al., LN density proved to be a stronger prognostic factor in patients with a LN count of ≥25 (HR 4.63) than in patients with a LN count of <25 (HR 1.62) [27]. Therefore, owing to interindividual variability in pelvic LN anatomy [40], LN density may not be a prognostic factor in patients with little lymphatic tissue. Furthermore, although greater numbers of LNs removed would most likely correlate with a more extended LN dissection, LN yield is intimately related to histological processing, and to the extent of pathologic review. Fifth, it is not known whether LN density determines survival any better than currently established pN categories of the TNM system [8]. Future studies are needed before LN density can be widely accepted as a staging system or used to replace pN staging. Finally, in the present study, only 2 reports used neoadjuvant chemotherapy; and therefore, it is not enough to determine whether LN density can be a valid marker for survival following neoadjuvant chemotherapy, which may favorably alter the nodal burden [8]. Additionally, given the use of neoadjuvant chemotherapy had gained increasing acceptance for treating invasive bladder cancer, the low rate of neoadjuvant therapy in this meta-analysis may have limitation on the generalizability. Further evaluation of the impact of neoadjuvant chemotherapy on LN density would be necessary.

Several limitations of this study should be considered. First, the HRs calculated in our meta-analysis may be overestimated, as many of the included studies obtained data retrospectively. Thus, adequately designed prospective studies are needed to obtain a more precise estimate. Second, the studies retrieved for our analysis were limited to those published in English, which may result in a language bias, although the present analysis does not support publication bias. Third, varying numbers of patients, median follow-up time, and quality scale might contribute to the heterogeneity of results for DSS found in this study. Although the random-effects model considers heterogeneity, and was used to analyze the studies with heterogeneities, the conclusions drawn from this meta-analysis should be approached with caution. However, heterogeneity of results for DSS was rigorously quantified and analyzed in our meta-regression and subgroup analysis, which contributes to a more reliable conclusion.

## Conclusions

In summary, the data from this meta-analysis indicate that LN density is an independent predictor of clinical outcome in LN-positive patients following radical cystectomy for bladder cancer. Although LN density may be related to histological processing and the extent of pathologic review, it is most likely a reflection of the quality and extent of pelvic LN dissection. LN density may be useful in future staging systems, thus allowing better prognostic classification of LN-positive bladder cancer following radical cystectomy. However, prospective validation would be required to define cut-off levels for LN density.

## Additional file

Additonal file 1:
**PRISMA 2009 checklist.**

## Abbreviations

LN: Lymph node
DFS: Disease-free survival
DSS: Disease-specific survival
OS: Overall survival
HR: Hazard ratio
CI: Confidence interval
TNM: Tumor-node-metastasis
